# Informal Providers—Ground Realities in South Asian Association for Regional Cooperation Nations: Toward Better Cancer Primary Care: A Narrative Review

**DOI:** 10.1200/GO.22.00260

**Published:** 2022-10-31

**Authors:** Prakash R. Nayak, Kunal Oswal, Conjeevaram S. Pramesh, Priya Ranganathan, Carlo Caduff, Richard Sullivan, Shailesh Advani, Ishu Kataria, Yogeshwar Kalkonde, Pavitra Mohan, Yogesh Jain, Arnie Purushotham

**Affiliations:** 1 Tata Memorial Hospital, Homi Bhabha National Institute, Mumbai, India; 2 Karkinos Healthcare, Mumbai, India; 3 Department of Global Health and Social Medicine, King's College London, United Kingdom; 4 School of Cancer and Pharmaceutical Sciences, King's College London, United Kingdom; 5 Bridge Medical Consulting, Delhi, India; 6 Public Health Centre for Global Non-communicable Diseases, RTI International, New Delhi, India; 7 Sangwari-People's Association for Equity and Health, Ambikapur, Chhattisgarh, India; 8 Basic Health Care Services, Udaipur, India

## Abstract

South Asian Association for Regional Cooperation (SAARC) nations are a group of eight countries with low to medium Human Development Index values. They lack trained human resources in primary health care to achieve the WHO-stated goal of Universal Health Coverage. An unregulated service sector of informal health care providers (IPs) has been serving these underserved communities. The aim is to summarize the role of IPs in primary cancer care, compare quality with formal providers, quantify distribution in urban and rural settings, and present the socioeconomic milieu that sustains their existence.

**METHODS:**

A narrative review of the published literature in English from January 2000 to December 2021 was performed using MeSH Terms Informal Health Care Provider/Informal Provider and Primary Health Care across databases such as Medline (PubMed), Google Scholar, and Cochrane database of systematic reviews, as well as World Bank, Center for Global Development, American Economic Review, Journal Storage, and Web of Science. In addition, citation lists from the primary articles, gray literature in English, and policy blogs were included. We present a descriptive overview of our findings as applicable to SAARC.

**RESULTS:**

IPs across the rural landscape often comprise more than 75% of primary caregivers. They provide accessible and affordable, but often substandard quality of care. However, their network would be suitable for prompt cancer referrals. Care delivery and accountability correlate with prevalent standards of formal health care.

**CONCLUSION:**

Acknowledgment and upskilling of IPs could be a cost-effective bridge toward universal health coverage and early cancer diagnosis in SAARC nations, whereas state capacity for training formal health care providers is ramped up simultaneously. This must be achieved without compromising investment in the critical resource of qualified doctors and allied health professionals who form the core of the rural public primary health care system.

## INTRODUCTION

Early cancer diagnosis and treatment are major global health issues that disproportionately affect low- and middle-income countries (LMICs). South Asia is one such region with eight countries that form South Asian Association for Regional Cooperation (SAARC), Afghanistan, Bangladesh, Bhutan, India, Maldives, Nepal, Pakistan, and Sri Lanka with low to medium Human Development Index values (except Maldives). SAARC countries have 25% of the world population and contribute to 25% of annual global cancer incidence and 16% of global cancer mortality.^1^ These numbers are likely an underestimate because of limited cancer and death registries. Delays in diagnosis and cancer treatment contribute to a significantly higher proportion of cancer deaths as compared with high-income countries.^1^ SAARC nations have a high-level Technical Committee on Health and Expert groups that are dedicated to improving outcomes in noncommunicable disease including cancer and maximizing human resources for health.^2^

**CONTEXT**

**Key Objective**
Who are informal health care providers, and why do people choose to seek care from them? How does their quality of care compare with prevailing standards, and can they contribute to the early diagnosis of cancer?
**Knowledge Generated**
More than 75% of health care across the rural landscape in South Asian Association for Regional Cooperation nations is provided by untrained informal providers. They are accessible and affordable but often have substandard quality when compared with the prevalent standards of formal health care.
**Relevance**
Although the training of increased numbers of formal health care workforce should be the priority, simultaneously acknowledging and upskilling informal health care providers may be a cost-effective and pragmatic bridge toward universal health coverage. Their network and social relationships with their local communities could be leveraged to create referral pathways for early cancer diagnosis. Health policy debates should consider involving informal providers in the care continuum and also ensure quality and accountability.

Minimizing delay in diagnosis of symptomatic cancer is associated with better survival, better quality of life, and lesser cost of treatment.^3^ The WHO strategy for Universal Health Coverage in SAARC has emphasized community-based primary health care as a core component. Reforming diverse health systems needs data on the nature, distribution, and quality of the primary health care workforce. The 2016 workshop of the World Organization of Family Doctors noted the substantial deficiencies of primary health care in SAARC nations, inadequate public systems, predominant private care, out-of-pocket payments, catastrophic health-related expenses leading to poverty, and reliance on alternative healers.^4^

As of 2014, the health expenditure in South Asia was 4% as compared with 10% of gross domestic product globally and 13% in the Organisation for Economic Cooperation and Development countries^5^ (Table 1). The modest investment in health of < 4% in all SAARC nations is made ineffective with emphasis on hospital-based and specialist care that relies on robust state capacity. SAARC nations have poor state capacity as reflected in the WHO workforce document.^6,7^ The number of medical doctors per 10,000 population ranges from 7.35 to 11 in the SAARC nations. The comparative figure for China is 22.2, that for the United States is 26.1, that for Germany is 44, and that for Sweden is 71.^8^ The scarcity of workforce and the urban-rural disparity are at the heart of delayed cancer diagnosis and are inadequately researched.

**TABLE 1:**
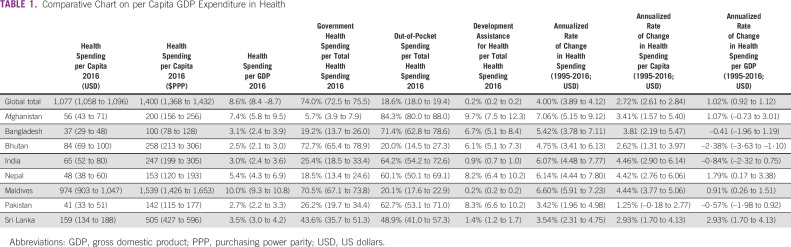
Comparative Chart on per Capita GDP Expenditure in Health

Two recent systematic reviews focused on delays and barriers to cancer care in low-income countries, including SAARC and the interventions performed to address some of these barriers.^9,10^ The lack of access was due to poor health literacy, stigma, poverty, loss of wages, lack of transportation, and geographical limitations. Other causes were inaccurate diagnosis, false reassurance, poor coordination of care, lack of referrals, and financial barriers. The interventions targeting health care professionals addressing these barriers reported only two studies conducted in SAARC (in Sri Lanka and Bangladesh).^10^ The WHO guide to cancer early diagnosis mentions lack of access to primary care as the step 1 barrier.^11^ None of the interventions reviewed access to primary health care. These systematic reviews highlighted the urgent need for studies that understand access to affordable and quality health care.^10^

Up to 75% of the primary care workforce in SAARC nations is informal.^12^ This workforce fills the gap created by lack of formally trained health care providers. The density, diversity, mobility, availability, education levels, composition, absenteeism, quality of the informal cadre, as benchmarked against formal workforce, and cost to system have not been adequately addressed in the mainstream health care literature.^13-17^

This narrative review aims to provide an overview of the supply side provider distribution: who the providers are, how they practice, variation in their quality, and the provider network that sustains this ecosystem.

## METHODS

An initial scoping exercise of the published literature on informal providers in primary health care in SAARC nations suggested that the data were too heterogeneous and/or sparse to allow a systematic review of qualitative studies as proposed by Dixon-Woods^18^ or a theoretical qualitative meta-synthesis as proposed by Sandelowski.^19^ We, therefore, performed a narrative review of the published literature in the English language from January 2000 to December 2021 using Informal Health Care Provider/Informal Provider, Primary Health Care as MeSH terms in the PubMed, Google Scholar, and Cochrane database of systematic reviews. All articles with the term Informal Health Care Provider/Informal Provider mentioned in the title or abstract were reviewed. Additional databases included those of the World Bank, Center for Global Development, American Economic Review, Journal Storage, and Web of Science. A manual search of other relevant articles and citation lists from the primary articles was performed. Gray literature in the English language that included policy blogs and allied publications was included. The review focused on papers in the SAARC nations that recognize the outsized presence and indispensable nature of informal health care providers, measured quality of care, the quantity of care provided as compared with formal health care providers, provider effort, interventions that attempt to upskill them, policy debates that have attempted to regularize and or regulate them, and themes that question the ethics of their existence and acceptance. The duration of the first two decades of the 21st century was chosen as this coincided with the 2001 UN sustainable development goals and directives to SAARC nations and the explicit focus on primary health care as key toward Universal Health Coverage.

## RESULTS

### Definitions and Scope

Informal providers or informal health care providers (IPs) form a vital cog in the wheel of rural health care ecosystem in SAARC nations. A systematic review published in 2013^12^ noted the absence of typology around informal providers. Since the informality of care is context-dependent, rigid criteria do not apply. A set of flexible criteria that include most informal providers entail those

1. Who have no formal institutional training
2. Who get paid by patients per transaction
3. Who do not have registration, regulation, or oversight by any institution.

It is important to note that these exclude community health workers who are trained and recognized by nongovernmental organizations and governments, for instance, the Accredited Social Health Activist woman workforce in India who are the first port of call for any health-related event.^20^ The World Bank data that measure community health workers at 0.514 per 1,000 in South Asia neglects IPs.^21^ Although human resources in health review for India published in 2011^22^ suggested that many of these IPs are registered medical practitioners, there is lack of evidence of any such registry across SAARC nations.

A list of workers that belong to this IP group is shown in Table 2.

**TABLE 2:**
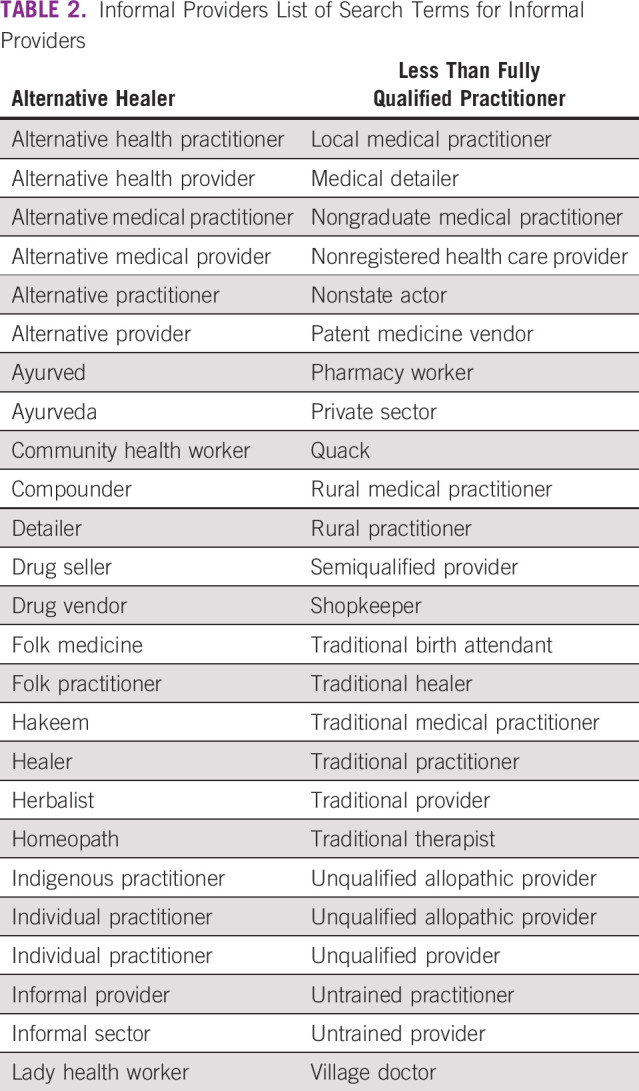
Informal Providers List of Search Terms for Informal Providers

The definition of an IP leaves them out of the ambit of conventional methods of measurement (registration, regulation, and oversight), leading to underestimation. Quality of care is inferred from crude descriptive statistics of referral, treatment, and success rates. The number of IPs is inferred from common disease subtypes, maternal and child health, reproductive health, malaria, and HIV/AIDS.

### Why Choose an IP?

There is a lack of trained formal doctors and state capacity to fill the gap lags by years^6^ (Table 3). Access is defined by the presence of trained doctors and not IPs. Universal health care hinges on the notion that patients prefer qualified public health care to informal private providers.^23^ The Medical Advice Quality and Availability in Rural India (MAQARI)^13^ study showed that patients chose both formal trained public providers and informal private providers for the same illnesses. The study showed that the difference in quality between an IP and an average public trained provider (standardized scores measured on the basis of knowledge using clinical vignettes and adherence to case-specific checklists) is sometimes small. An IP from a relatively richer state in southern India (Tamil Nadu) is sometimes better than a trained provider in some of the poorer states in north/central India (Bihar).^13^

**TABLE 3:**
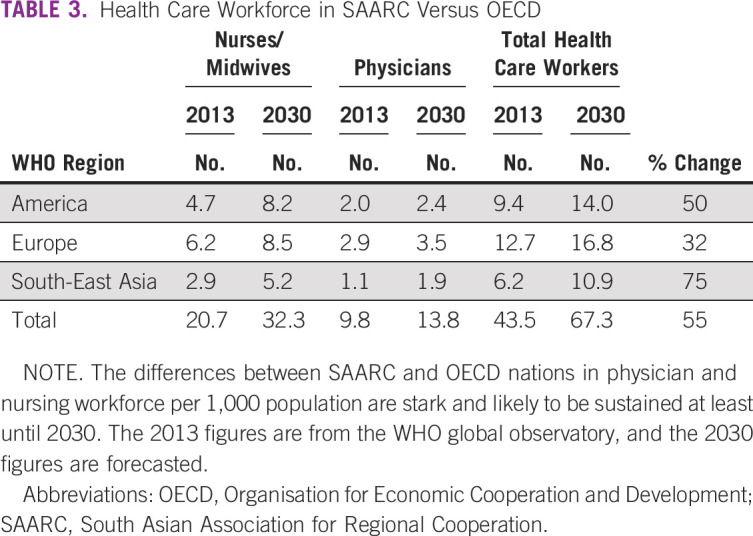
Health Care Workforce in SAARC Versus OECD

Proximity to households in both rural and urban settings leads to cost savings by avoiding travel and loss of daily wages.^24^ Formal care may be 5-15 times more expensive as compared with informal care.^25,26^ IPs provide drugs cheaper than pharmacies by repackaging drugs in smaller units.^24,27^ IPs belong to the locality they serve, which may build trust and accountability and may help address social and cultural barriers as they understand local customs and practices. IPs allow flexible charges, deferred payments, accommodating financial insecurity, and lack of insurance. The longevity of association sustains a stable relationship compared with attrition and absence common in public clinics.

### Measuring Quality of Care

The quality of care given by a provider varies in each transaction, cannot be generalized, is difficult to measure, and differs on the basis of the illness and provider training. The methods used for measuring quality are standardized patients, medical vignettes, and direct observation. Other reported dimensions of quality are based on technical aspects of adhering to existing national guidelines and referral pathways. This has limitations when applied to SAARC nations as guidelines and referral pathways are often absent, and when present, they are poorly disseminated and provider awareness and knowledge may be poor.^12^ A systematic approach to adapt and contextualize international guidelines is underway in India.^28^ This pragmatic approach allows a framework to alter guidelines to suit local needs, affordability, and availability of resources.^28^

In a large country like India, there is a huge geographic variance in the competence of IPs. The accuracy of diagnosis in the best performing states like Tamil Nadu and Gujarat for eclampsia, tuberculosis, and dysentery was 94%, 93%, and 91%, respectively. The figures for the worst performing states (Jharkhand, Bihar, and Uttar Pradesh) were 12%, 5%, and 16%, respectively.

In a study performed in rural Madhya Pradesh, India, 67% of health care providers did not have any medical qualifications.^17^ Even at public health facilities, 63% of the care was provided by staff who had no medical training. This was due to chronic understaffing and absenteeism among qualified staff. Studies performed in urban and rural India and a similar study from China had surprisingly similar results, despite the large difference in the gross national income per capita ($583 US dollars (USD) in Madhya Pradesh and $3,179 USD in Shaanxi, China).^29^ The average time spent per patient was < 2 minutes, < 20% of the necessary questions were asked for emergent and nonemergent needs, only half the patients had close to correct treatment, and less than one fourth had correct diagnoses.

### Provider Effort

**FIG 1:**
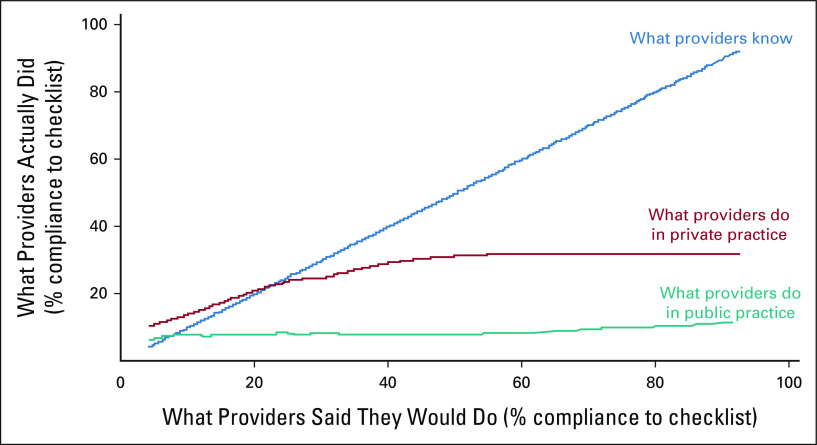
The know-do gap in medical care. The horizontal axis plots what a provider knows, as measured by medical vignettes, using percentage compliance with a medically necessary case-specific checklist of history questions and examinations (by rotating the curve). The vertical axis plots what the provider actually did with a similar patient, observed in practice. Every history question and examination can be compared in a pairwise comparison. If providers did everything they told us they would do, we should observe them on the 45° line. At very low levels of knowledge, practice is constrained by knowledge; at higher levels of knowledge, there is a significant gap between knowledge and practice; the know-do gap is larger in the public sector (in which there is no correlation between practice and knowledge), but even in the private sector, there is a significant gap at higher levels of knowledge. Used with permission of Annual Reviews, Inc, from Annual Review of Economics, Quality of Primary Care in Low‐Income Countries: Facts and Economics, Das et al, 6:525‐553, 2014; permission conveyed through Copyright Clearance Center, Inc.^14^

Studies have shown provider effort to be a critical factor for better quality care. The effort is measured as a know-do gap between what providers know and the measured facts of patient interaction (what they do). Competence is measured by standardized scores using medical vignettes and performance by observing them in practice and measuring adherence to checklists. Das and Hammer^14^ in the MAQARI study hypothesize that a 45° slope of correlation between knowledge and effort would imply that providers do what they know. This is observed at the lowest levels of competence, but as knowledge and training gets better, a larger gap is seen between what one knows and what one does (Fig 1). The gap is worse in the public sector, but remains large in the private sector too. Health policy frameworks attribute this to overburdening or lack of equipment or infrastructure.

Das et al^30^ in the 2013 study measured this phenomenon in rural Madhya Pradesh, India, among public practitioners, 83% of whom also had private clinics. The know-do gap was less in the private practice than in public clinics. The *same* providers spent more time, reached more accurate diagnoses, and aligned better treatment.^30^ This could imply effects of financial incentives on better effort in the private sector.

The administrative accountability that drives incentives in the public sector is seldom enforced. A strong incentive for poor patients is the availability of subsidized or free drugs in the public sector. This leads to a curious effect of over prescription for financial gain in the private sector and over prescription in lieu of care in the public sector.^31^

Leonard et al have measured the role of nonfinancial incentives^32^ in increasing motivation for better care. When the provider was aware that patient transactions were monitored for compliance and quality, an immediate high and a sustained improvement was noted (Hawthorne effect). These findings have been replicated across LMICs and high-income countries. Bjorkmann and Svenson used this effect in rural Uganda by employing community workers to improve local accountability of the public providers.^33^ A reduction in the under-five mortality (33% improvement one year into the program) was seen in the experimental villages without effort in improving training or infrastructure.

The experience from national tuberculosis control programs in India suggest that IPs who are trained perform well with triage, early referral, leading to better outcomes in patients with tuberculosis.^34^ These findings may be applicable for improving early diagnosis of both symptomatic and asymptomatic cancers.

### Mapping the Types and Number of Informal Providers

A systematic mapping of the network of these providers is lacking. The MAQARI study has the largest nationwide data across 19 Indian states, which is representative of 90% of rural households. Few similar studies have been performed outside of India, notably in Ghana,^35,36^ Zambia,^37^ Nigeria,^38^ Bangladesh,^39,40^ and Nepal.^41^

A brief summary of the MAQARI study describes the landscape that is similar across SAARC.^13^ The study notes that of the 1,519 Indian Villages surveyed, 74% had at least one health care provider and 86% of providers worked in the private sector. Of the 3,473 providers surveyed, 68% (2,367) were IPs in the private sector, 24% (842) belong to the AYUSH cadre (Ayurveda, Unani, and Homoeopathy degrees; formal providers trained in alternative nonallopathic streams), and only 8% (264) had an MBBS. degree. The average village (range 1,000-5,000) had 5.4 IPs. The proportion of these providers did not change significantly in more developed states (as per the socioeconomic index). The systematic review by Sudhinaraset et al^12^ from 2013 shows similar high rates of IPs across countries, 65%-77% in Bangladesh, 55%-77% in Thailand, 77% in Uganda, 36%-49% in Nigeria, and 33% in Kenya. The summary of the MAQARI study estimates this number at 80% for India.

### Low Quality of Care and Diagnostic Delay: Causal Links

The WHO guide to cancer early diagnosis framework states that access to care, evaluation of disease, and referral for subsequent care are the three pivotal steps to good cancer outcomes. Access to some form of health care is near universal in SAARC nations. It is the substandard quality of care that undermines the utility of this access. Tertiary care referral institutes see a huge proportion of patients with untreated or improperly treated cancer with disease at an advanced stage. The key barriers to early diagnosis of cancer as shown in a systematic review^9^ include health illiteracy; cancer stigma; lack of access to reliable primary care; inaccurate diagnoses; limited access to diagnostics; poor coordination of care; and geographical, financial, and sociocultural factors. This poor coordination of quality of care is the causal link that mediates inaccurate diagnoses and poor referral, leading to advanced stage at presentation, which worsens mortality, thereby also feeding into the stigma surrounding cancer. Of the more than 300 studies that reported delay and barriers to early cancer diagnosis, only 25 studied interventions. Furthermore, very few described the prominent role played by IPs.

The success of interventions addressing barriers to delayed diagnosis had 12 of 25 studies performed in groups of health care professionals, including doctors, nurses, midwives, dental professionals, and student health care professionals. However, none of these studies included IPs whose proportion outnumbers the formal health care worker by a factor of 3 or higher in most LMICs.^12^

### Publicly Funded Primary Health Care and Informal Providers: Policy Implications

There is an implicit acceptance that IPs are part of the health care fabric of SAARC nations. A national sample survey of India in 2014 showed that 70% of patients visited a private health care worker as the first point of contact. Studies show that a majority of these are IPs.^17,42-44^ Surveys in their current format are unable to distinguish formal and informal health care providers, and this potentially misleads policy makers. Acknowledgment and measurement of this workforce is essential. Under the Indian Medical Act 1956, it was illegal for anyone to practice medicine and dispense drugs without a university medical qualification. The current National Medical Commission Act of India makes provision for temporary licenses to community health providers for primary and preventive care. The exact nature of these regulations and scope of licenses are unclear. Any specter of regulation from powerful governmental agencies is construed as a threat to existence. The establishment worries that legitimizing them is akin to promoting illegal activity. As of today, enforcement of regulations and punitive actions is not feasible because of lacking state capacity and strong support from local communities and local politicians. Recognizing this, many Indian states under the federal health structure, including West Bengal, Andhra Pradesh, Bihar, and Chhattisgarh, sought ways to train these cadres as community health care workers or paramedics.^45-47^ IPs have participated in polio vaccination drives, tuberculosis treatment programs, UNICEF's social mobilization programs, mother and child health programs, and distribution of contraception. Das et al^13^ note that IPs do better with triage, diagnosis, and treatment checklists because of higher effort than some formal providers. Governments recognize the value in involving them in a sustained, trained manner for emergency services, triage, referrals, public health awareness, and screening programs. National Academies of Sciences, Engineering, and Medicine (2018) has suggested integrating IPs into formal networks as paramedics. The Health Ministry of India in its 2019 document has recognized this and has heightened efforts in employing nonmedical, mid-level health care providers at sub-centers, Health and Wellness Centres (HWC). A systemic change included a curriculum of bridging courses for nurses and AYUSH doctors, Bachelor of Science in community health, and 3-year diplomas planned by state governments of Assam and Chhattisgarh. Many of these noble intentions even among semitrained/trained cadres have been met with legal roadblocks from the allopathic medical community. These challenges may be worse with the IP cadre. The sector of IPs is emergent and is unregulated and has a wide variance of quality of care delivered. Acknowledging, upskilling, and integrating such that they work alongside the formal, trained health care workforce within the health care ecosystem is likely to improve quality of care and accountability.

Central, state, and private medical colleges, currently, do not have the capacity to train sufficient MBBS graduates. While the system catches up, countries need urgent interim measures to formalize this huge cadre of health care workers who serve patients.^45,47,48^ Das et al^49^ in the randomized trial for multitopic training of informal health care workers conducted in West Bengal, India, showed improved case management without worsening tendency to violate norms or worsen performance in clinical practice. They concluded that upskilling IPs may be an effective short-term strategy to complement the ailing health care system. Before this trial that focused on upskilling a broad range of tasks that are routinely performed by IPs in primary health care, randomized trials showed improved performance in specific tasks such as treatment of malaria,^50^ HIV-focused care study in Pakistan,^47^ care for sexually transmitted illnesses in Peru,^51^ and training pharmacists in correctly treating cases of urethral discharge.^51^ Das et al make a compelling argument that at an annual salary of $6,000 USD, the government could hire 11 MBBS doctors and the same amount could train 360 IPs yearly who were within 5 km of an average household. This annual salary is not reflected in the additional infrastructural, staffing, and pension-related costs that governments have to bear. A systematic scoping review in LMICs^34^ noted the positive impact on outcomes of employing IPs after support and training in detection, diagnosis, and administering antituberculous therapy. A similar policy focusing on training IPs to detect signs of early cancer and encourage referral to the nearest known cancer center may improve outcomes. The high-level meetings of the SAARC Technical Committee on Health and Population activities could be the focal point for consideration and debate on this issue.

The continuous focus on funding and improving services at rural primary health care centers (PHCs) and HWCs is vital. The 2022 audit report conducted in 18 states in India noted increased satisfaction levels, reduced out-of-pocket expenditure, and a move from private unqualified healers to qualified HWCs.^52^ This is in line with the gradual replacement of traditional birth attendants with institutional health care delivery of pregnant women over the past two decades in LMICs.^53^

The ambitious national project to operationalize 200,000 HWCs in India to strengthen the rural primary health care infrastructure was expected to have labor deficits. The 2022 audit report also noted that most states have failed to employ the full range of workers at PHCs and HWCs.^52^ These include nurses, pharmacists, and ancillary health care workers. IPs could be incentivized to fill this gap and align with the goals of PHC within the formal health care ecosystem.

### Limitations

We recognize the limitations of this narrative review as studies were not objectively selected on the basis of methodological quality. However, the studies in this review had methodological rigor, large sample size, and standard-of-care metrics for evaluation of qualitative and quantitative aspects of health care provision. The conclusions matched the real-world scenario, and estimates have remained robust over a decade. The demography of the populations and the shared cultures allows a safe extrapolation for a policy recommendation across SAARC. We recognize the social, political, and legal challenges of acknowledging informal providers. The system-wide changes that follow the act of acknowledgment are unclear. Regulating an emergent space runs the risk of destroying its fabric as it may create entry barriers and special interest groups. As the economy improves, capacity for producing expertise and demand for better care will increase and the space for informal providers will inevitably shrink.

There remain systemic concerns about the perverse financial motives and harmful, wasteful practice of some IPs. However, they continue to thrive albeit in smaller numbers in states and nations that have a high coverage and proficiency in rural primary health care (Tamil Nadu in India, Sri Lanka, sand Bangladesh). The WHO Advisory Group under the Health System and Governance department suggested aligning private for-profit and not-for-profit actors to the public sector.^54^ Engagement of IPs may foster accountability and enable the rural population as the key stakeholder to bridge and maximize services provided by either sector. This is vital as the incentive structure, motivation, and moral high ground of those who commit to the public rural health care system are tenuous. It must be valued and preserved. The funding for the formal health care provider sector should be a higher priority and must not compete with the regulatory or upskilling needs of the IP sector. The key challenge for informed policy is lack of information and systematic research. Governments must sponsor studies to understand and measure the impact that IPs and other for profit systems have on rural health care practice and delivery.^55^

## DISCUSSION

In conclusion, SAARC nations have a plethora of informal providers forming the base of the health care worker pyramid. They provide options to people who have poor access to formal health care systems. They have deep local roots, strong social connections, and established networks of care that covers a majority of remote villages. This sector is emergent and unregulated and has a wide variation in the quality of care delivered. Available but poor care worsens health outcomes. Integrating these providers into the mainstream is controversial with no consensus. Strict regulation, which disallows IPs, may reduce some harmful care, but will leave a segment of the population with no access to health care. The state capacity for such regulation is lacking. Acknowledging the IPs and frequent training may improve equity, quality, and accountability of care. It will be complementary to parallel efforts to improve the number and quality of doctors, nurses, and other formal health care workers. Training IPs for general education, cancer screening, early identification of symptomatic cancers, prompt referral to subdistrict or district hospitals for workup, and sensitization about palliative care is an urgent unmet need. The Lancet Commission^56^ for sustainable development goals for high-quality health systems outlined four values: that systems are for people, equitable, resilient, and efficient. The network of IPs may satisfy these values as building blocks. This network must complement and align with the needs of the public rural health care ecosystem, allowing the public system to hold them accountable.
